# The first emerging records of a dragonfly in the dark zone of subterranean ecosystems: Exuviae and newly emerged adults of Hyrcanian Goldenring, *Cordulegaster vanbrinkae* in Danial Cave, northern Iran

**DOI:** 10.1002/ece3.70157

**Published:** 2024-08-09

**Authors:** Pouria Ghelich Khani, Mohsen Kiany, Ali Turk Qashqaei

**Affiliations:** ^1^ Department of Biodiversity and Ecosystem Management, Environmental Sciences Research Institute Shahid Beheshti University Tehran GC Iran; ^2^ Zoology Section, Department of Biology, School of Science Shiraz University Shiraz Iran; ^3^ Borderless Wildlife Conservation Society Tehran Iran

**Keywords:** cave fauna, darkness, endemic species, Hyrcanian Forest, Odonata, riverine cave

## Abstract

Riverine caves are special habitats that are home to many aquatic and terrestrial species. Some Odonata species and their emerging are recorded at the entrance and in the twilight zones of subterranean habitats around the world. However, the emergence of any Odonata species has not been recorded in the dark zones of caves or other subterranean habitats. We report the first evidence of the emerging of the Hyrcanian Goldenring, *Cordulegaster vanbrinkae* Lohmann, 1993, as an endemic species of the Hyrcanian biogeographical region, in the dark zone of Danial Cave, in the World Heritage‐listed Hyrcanian Forests, northern Iran. During 2020–2023, three newly emerged and three exuviae of the species were recorded in the entrance zone (25 m) and the dark zone of the cave (200–280 m). The main hypothesis of the study is the entry and exit of adults from the cave entrance. However, we still do not know if the newly emerged will leave the cave or not. We still need more study on the biology and ecology of the species inside and around the cave. Danial Cave, with its high biodiversity, is one of the most important caves in the Middle East, and is urgently in need of conservation as a national natural monument.

## Graphical abstract

The paper is about the first emerging records of an Odonata species in the dark zones of subterranean ecosystems. According to the scientific literature, the records are new for the order Odonata. Also, the study considered some behaviors of Hyrcanian Goldenring, Cordulegaster vanbrinkae in darkness.

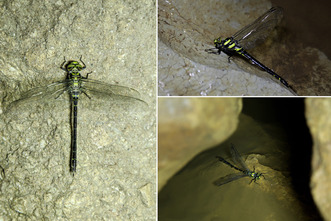

## 1 INTRODUCTION

Riverine caves are important for the breeding of some semiaquatic insects such as Plecoptera, Ephemeroptera, and Trichoptera, which spend a part of their life cycle in the dark zone of these subterranean habitats (Pacheco et al., 2021; Romero, 2009). However, until the 1990s, records of species of the Order Odonata from subterranean or cave habitats were extremely rare (Thompson & Kiauta, 1994), and after that, most recorded cases are considered accidental and occurred by chance in these habitats (Kiauta, 1995; Krieg‐Jacquier & Sansault, 2016; Manenti et al., 2013). In some studies, the presence of the Odonata species at the entrance and in the twilight zones of natural or artificial subterranean habitats is mentioned by Thompson and Kiauta (1994), Manenti et al. (2013), and Krieg‐Jacquier and Sansault (2016).

There are over 100 identified Odonata species in Iran, but no species have been recorded from subterranean habitats in the country to date (Boudot et al., 2021; Schneider et al., 2018; Schneider & Ikemeyer, 2019). The Hyrcanian Forest, World Heritage‐listed, is the refuge for three endemic Odonata species: The *Aeshna vercanica* Schneider, Schneider, Schneider, Vierstraete & Dumont, 2015; the *Coenagrion australocaspicum* Dumont & Heidari, 1995; and the Hyrcanian Goldenring, *Cordulegaster vanbrinkae* Lohmann, 1993 (Boudot et al., 2021; Holuša et al., 2015).

Today, the Hyrcanian Goldenring is a well‐known species and is recorded in Iran, Armenia, and Azerbaijan (Boudot et al., 2021; Holuša, 2015, 2022; Holuša et al., 2015; Schneider et al., 2021). The species is listed as “Data Deficient” on the “IUCN Red List,” because of a lack of information about their ecology, status and threats (Boudot, 2006; Smith et al., 2014). The Hyrcanian Goldenring is a shade‐friendly species and occurs along shaded brooks and rivulets in the Hyrcanian Forest, in the Caspian region (Holuša et al., 2015; Ikemeyer et al., 2015). Herein, 30 years after the first description of the Hyrcanian Goldenring, we present the first records of the species in Danial Cave and the first emerging records of an Odonata species in the dark zone of any subterranean habitat.

## 2 METHODS

### 2.1 Study area

**FIGURE 1:**
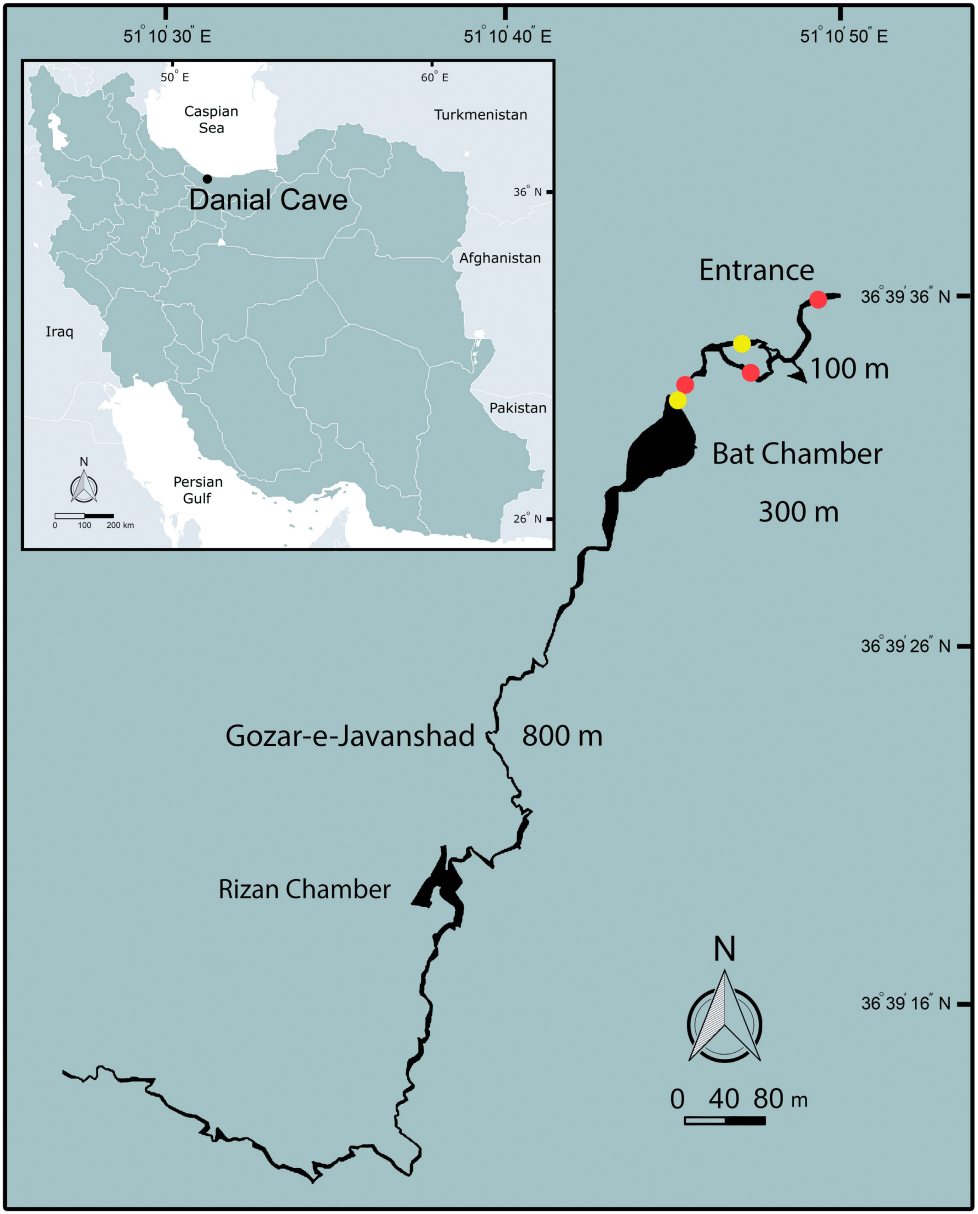
Locations of Hyrcanian Goldenring, *Cordulegaster vanbrinkae* Lohmann, 1993 in Danial Cave: Newly emerged adults (yellow point) and exuviae (red point).

**FIGURE 2:**
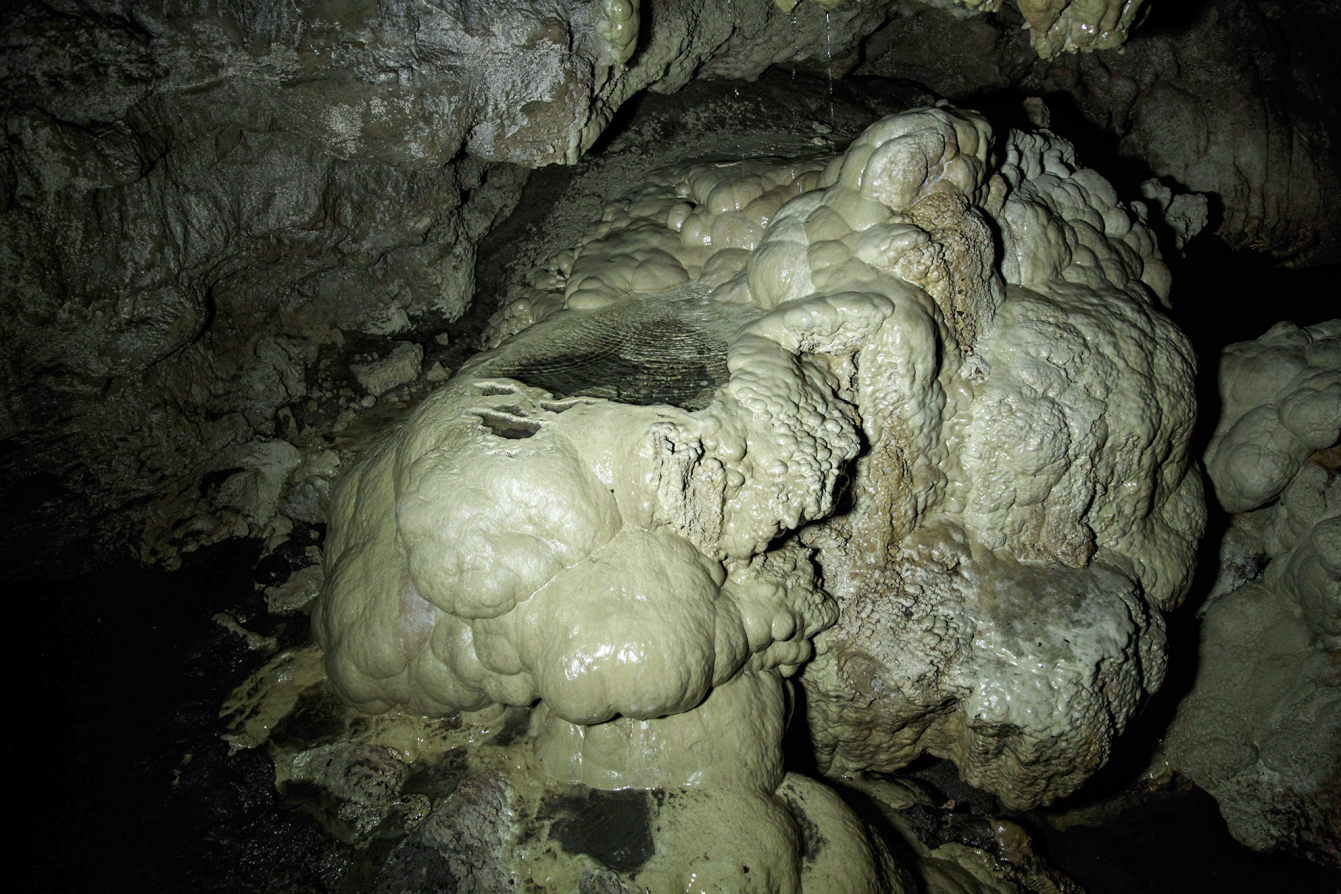
A karstic structure in the Danial Cave around 200 m from the cave entrance, near the first and fifth records, after a heavy rain, Feb 2020, © Pouria Ghelich Khani.

**FIGURE 3:**
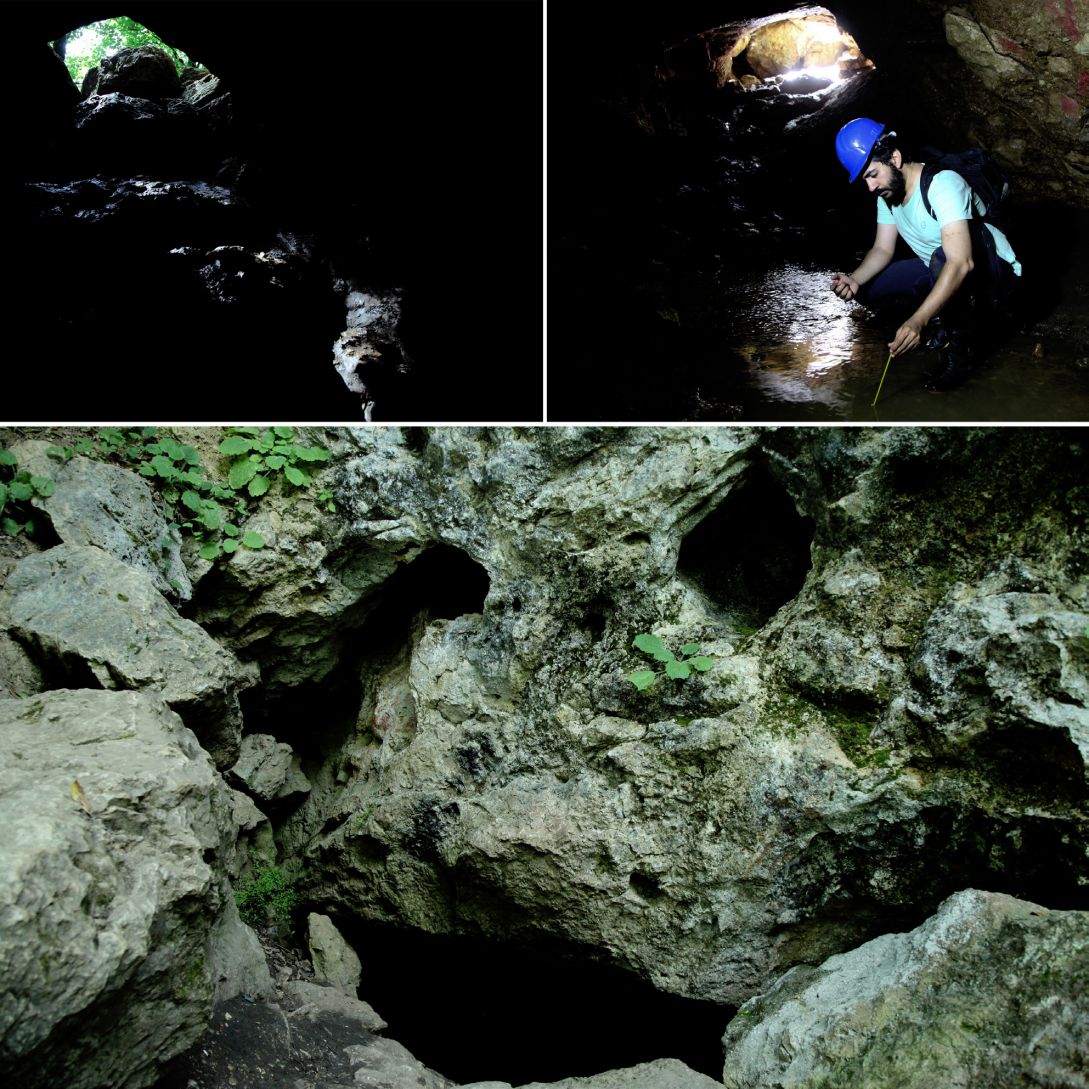
Three views of Danial Cave entrance: Up‐right: A view from inside with collecting water temperature data, 2020; up‐left: Another view from inside, 2024; down: A view of the cave entrance from outside. © Pouria Ghelich Khani.

**FIGURE 4:**
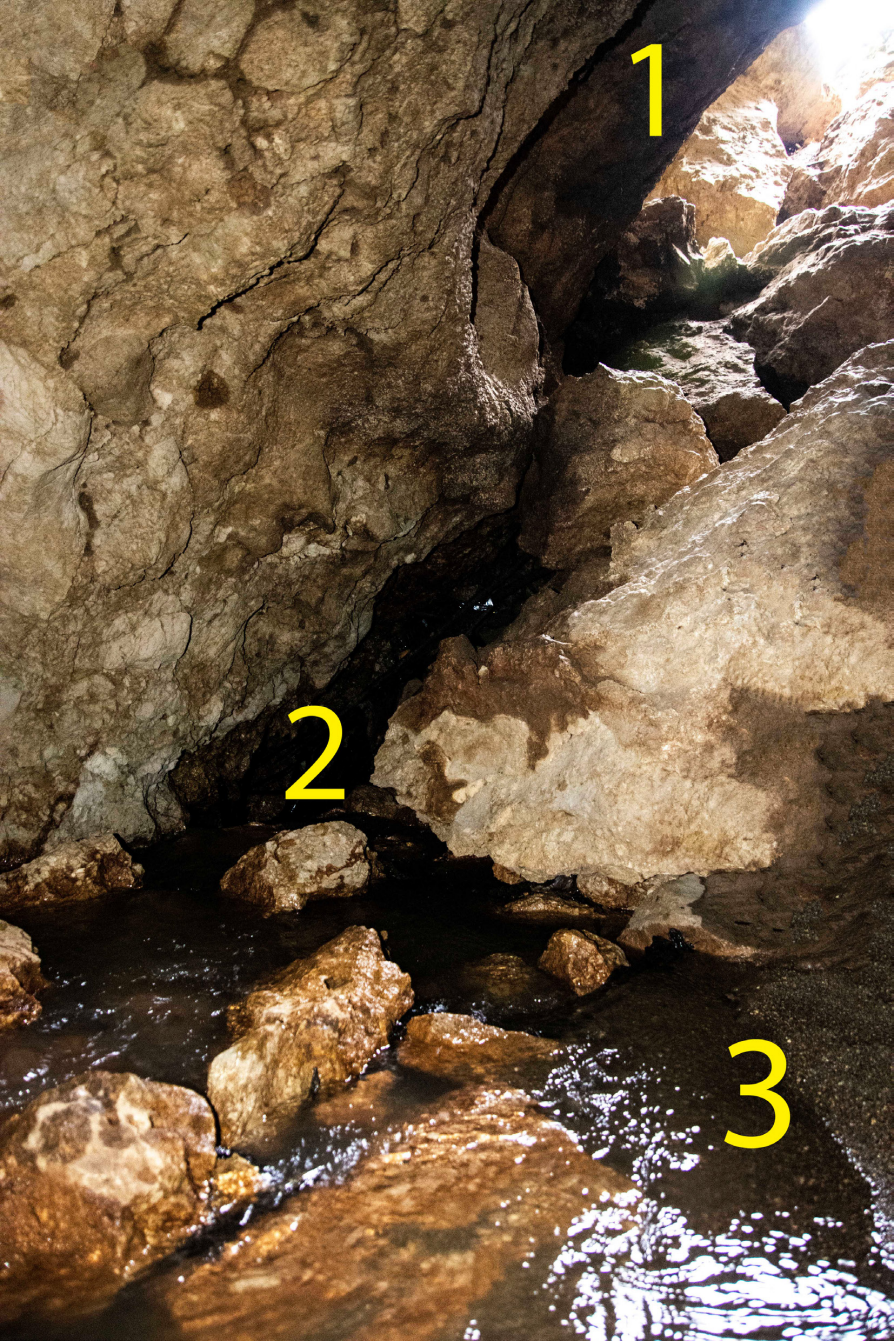
Crucial ecological parts of Danial Cave for the Hyrcanian Goldenring, *Cordulegaster vanbrinkae*: (1) the cave entrance; (2) water exit point; and (3) stream in the cave bed.

**FIGURE 5:**
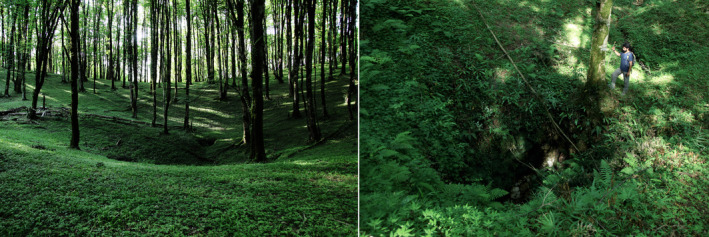
Left: A view of the shaded Hyrcanian Forest, with a sinkhole in the middle of picture. Right: A close view of a sinkhole in upper elevations of Danial Cave. © Pouria Ghelich Khani.

Danial Cave (36.660072° N, 51.180949° E, 175 m above sea level) is located near Danial village, Salmanshahr county in Mazandaran province, northern Iran (Figure 1). The distance from the cave entrance to the cave bed is about 7.5 m, with a 45‐degree slope. The cave has an oval‐shaped entrance (longer diameter c. 360 cm, shorter diameter c. 85 cm) with several sinkholes. It is a karstic riverine cave and is considered one of the longest riverine caves in the Middle East (2158 m, Figures 1, 2, 3, 4, 5). The air temperature in the warm and cold seasons inside the cave was 17 and 14°C, respectively. The water temperature in the same seasons inside the cave was 16 and 12°C, respectively (Figure 3); the discharge of the cave stream was 0.5 m^3^ per second in May 2024. According to Ghelich Khani et al. (2023), identified vertebrate species in the cave include the Greater Horseshoe Bat, *Rhinolophus ferrumequinum*, Blasius's Horseshoe Bat, *Rhinolophus blasii*, the Lesser Mouse‐eared Myotis, *Myotis blythii*, the Hyrcanian Field Mouse, *Apodemus hyrcanicus*, the Hyrcanian Wood Frog, *Rana pseudodalmatina*, and the Eurasian Marsh Frog, *Pelophylax ridibundus*. Also, cave crickets (Rhaphidophoridae), mayflies (Ephemeroptera), endemic amphipods (*Niphargus daniali*), endemic springtails (*Plutomurus danialensis*), and freshwater crabs (*Potamon ibericum*) were recorded in the cave (Barjadze et al., 2024; Esmaeili‐Rineh & Sari, 2013; Ghelich Khani, 2017). The cave has been damaged by many visitors, and parts of it have been destroyed or altered (Ghelich Khani et al., 2023).

### 2.2 Data collection

Field work to identify fauna in the cave was conducted from July 2013 to Aug 2023. We carried out a total of 58 surveys in the study period for seasonal monitoring of fauna. Moreover, approximately 9 h (range 3–21) are spent on each survey. Furthermore, the specimens were photographed, and their exuviae were collected.

## 3 RESULTS

**FIGURE 6:**
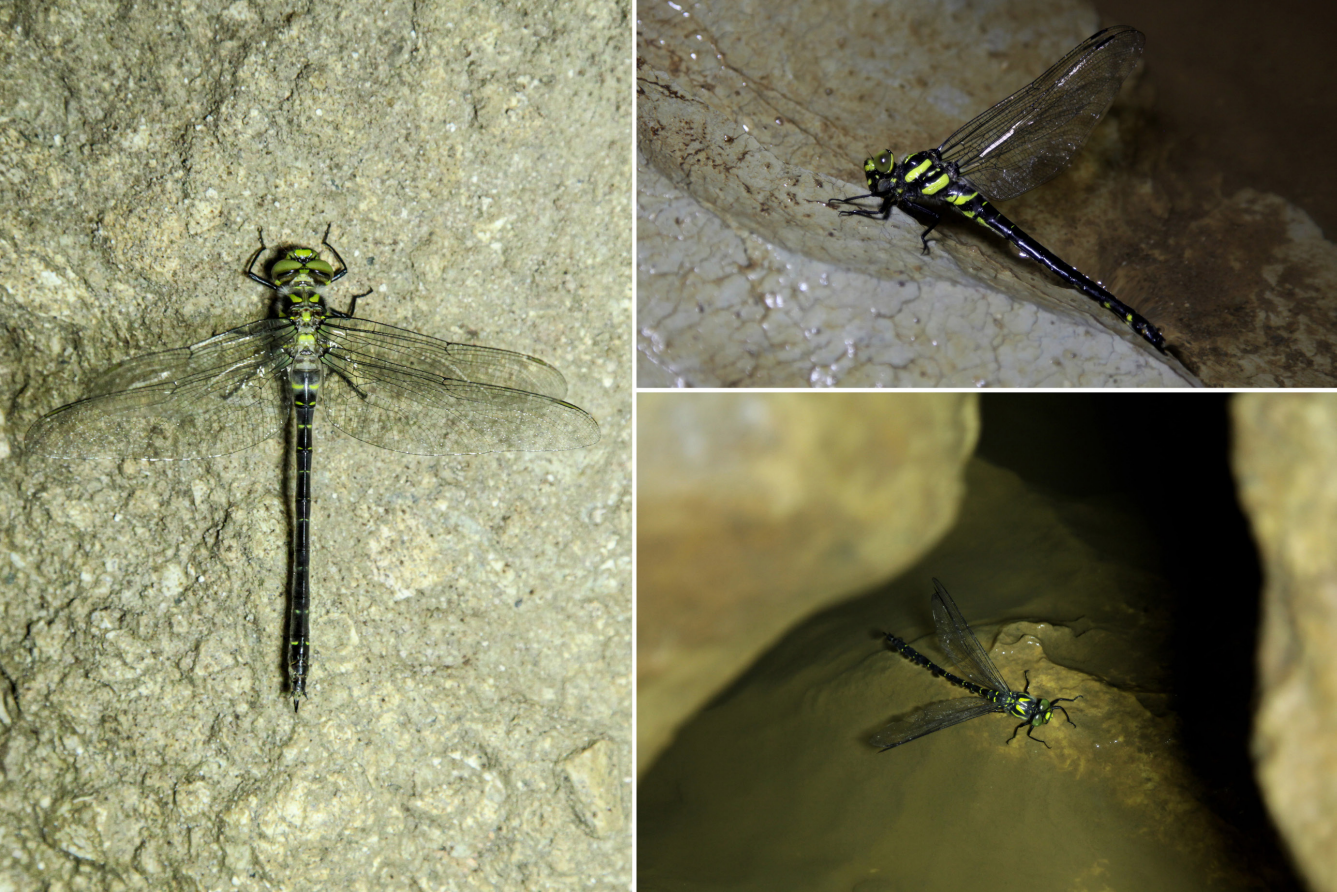
Three newly emerged adults of the Hyrcanian Goldenring, *Cordulegaster vanbrinkae* Lohmann, 1993 in the dark zone of Danial Cave. © Pouria Ghelich Khani.

**FIGURE 7:**
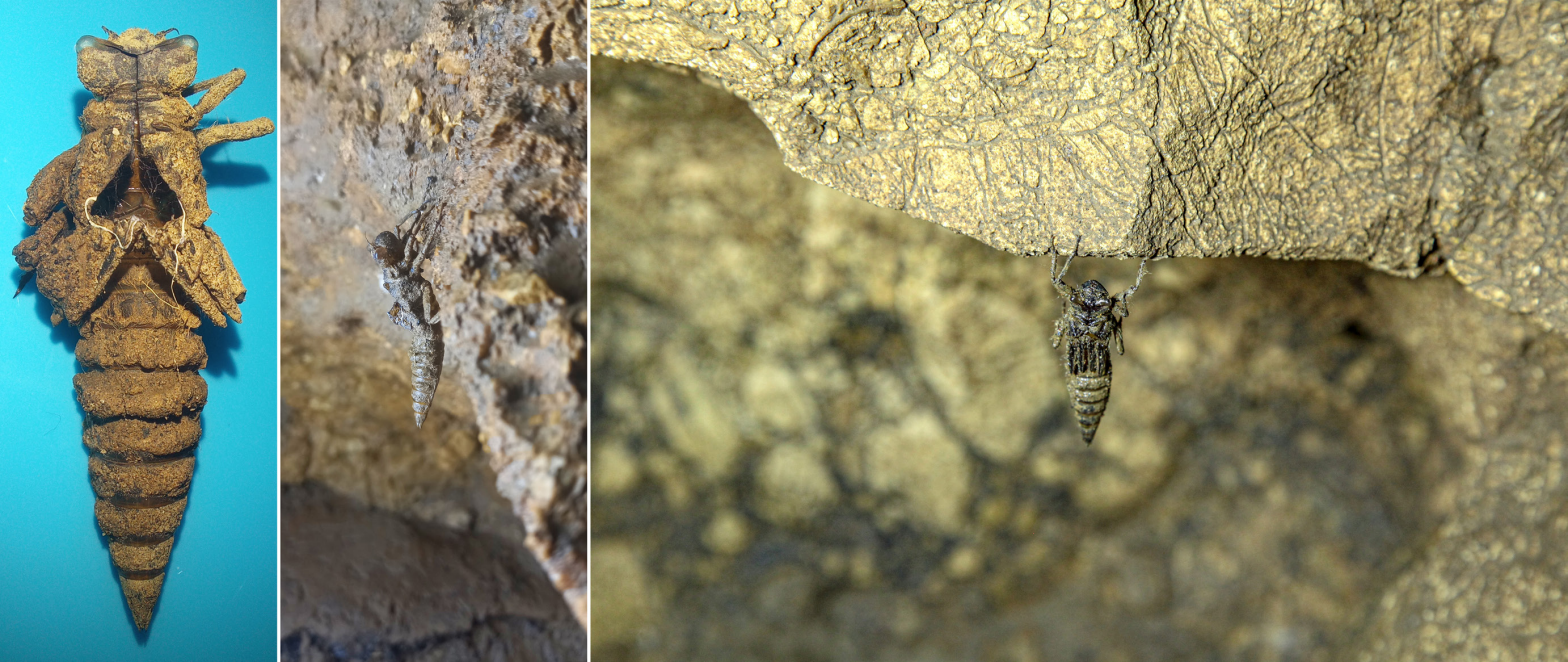
Three exuviae of the Hyrcanian Goldenring, *Cordulegaster vanbrinkae* Lohmann, 1993 in Danial Cave. © Pouria Ghelich Khani.

In total, three newly emerged and three exuviae of the Hyrcanian Goldenring were found in 25 m (the entrance zone) and 200–280 m (the dark zone) from the entrance of the Danial Cave during 2020–2023 (Table 1, Figures 1, 6, and 7). The findings are the first records of an Odonata species emerging in the dark zone of these habitats.

**TABLE 1:** Newly emerged adults and exuviae records of the Hyrcanian Goldenring *Cordulegaster vanbrinkae* Lohmann, 1993 in the dark zone of Danial Cave during 2020–2023.

No.	Height from water surface (cm)	Distance from the cave entrance (m)	Stage	Sex	Record date
1	196	200	Exuvia	Male	9 June 2020
2	98	280	Exuvia	Male	9 June 2020
3	95	280	Newly emerged adult	Female	9 June 2020
4	15	280	Newly emerged adult	Female	17 June 2020
5	10	200	Newly emerged adult	Female	18 June 2021
6	21	25	Exuvia	Female	13 June 2023

In addition, we found *P. danialensis*, *N. daniali* and Ephemeroptera larvae as potential food items for the larvae of Hyrcanian Goldenring in Danial Cave. Moreover, *P. ibericum* and frog species are not potential aquatic predators for the larval stage because they are absent in the dark zone (200–280 m), so the cave is a relatively safe place for the larval stage. However, the potential predators of the adult stage of the Hyrcanian Goldenring are bats in the Cave. The presence of *C. vanbrinkae* in Danial Cave has advantages and disadvantages for them (Table 2).

**TABLE 2:** Costs and benefits of Danial Cave as a refuge for the Hyrcanian Goldenring *Cordulegaster vanbrinkae* Lohmann, 1993 in northern Iran.

Costs, as an ecological trap	Benefits
Cooler in the warm season Wandering adults in darkness Critical for emerging (i.e., darkness and bat species as predators of adults) Increasing cannibalism Human disturbance	Absence of aquatic predator in the dark zone Availability of aquatic preys throughout the year Stable natural environment The possibility cave leaving

## 4 DISCUSSION

### 4.1 Entry scenarios of Hyrcanian Goldenring into the cave

**FIGURE 8:**
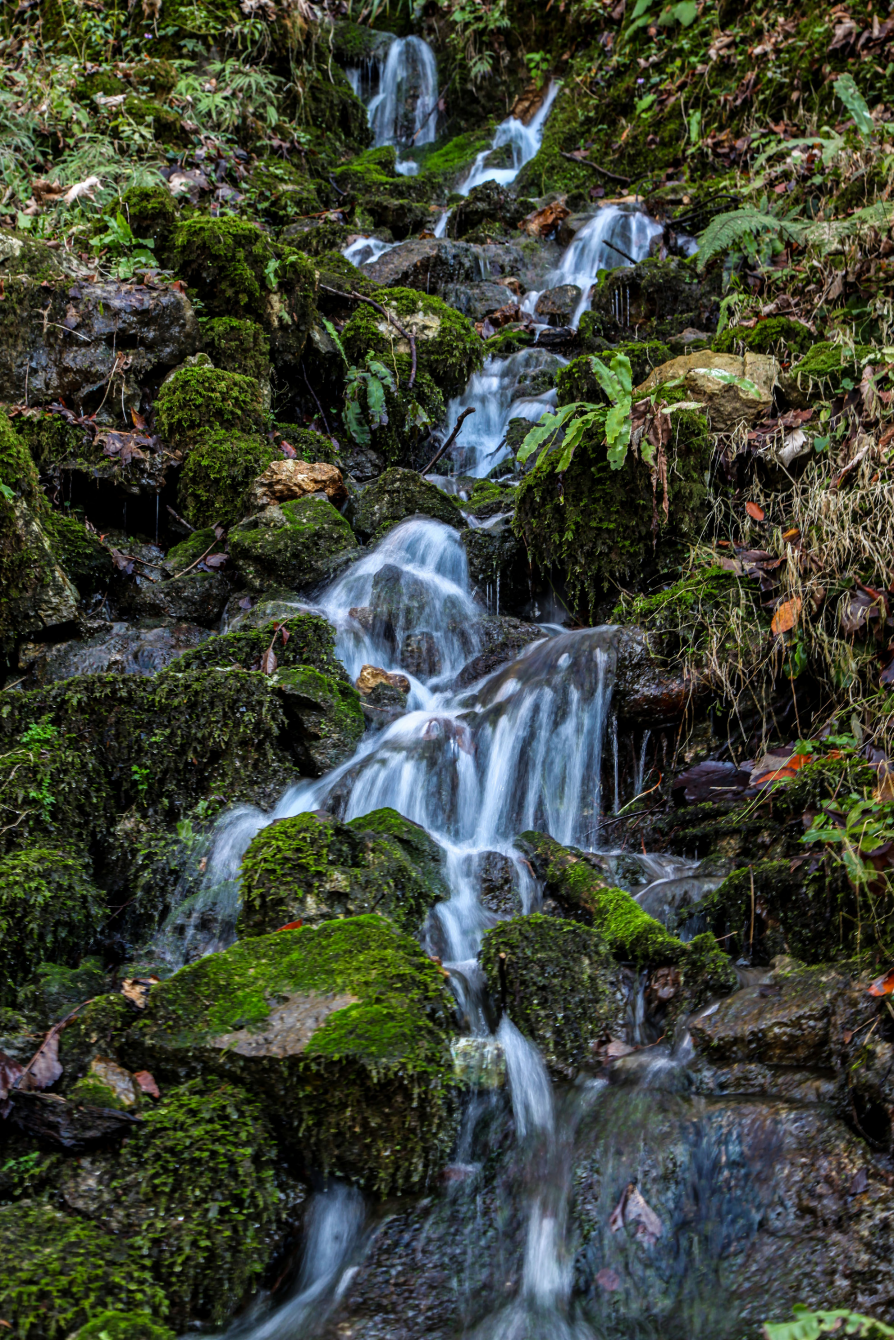
A view of waterfalls outside the cave. Sep 2020. © Pouria Ghelich Khani.

In our case, there are three scenarios of species entry in Danial Dave. The main scenario is the entry of female adults from the cave entrance and intentional or accidental oviposition. The secondary scenario is intentional or accidental penetration or entering of larvae in the cave from the water exit point against the flow (Figure 4). In this case, after passing more than 30 m, the larvae reach the stream with several waterfalls (Figure 8, more than 50 cm) outside and inside the cave and disperse and move up to 280 m into the cave. This scenario seems nearly impossible due to the many barriers. The third scenario is the penetrating of larvae from sinkholes. The possibility of the larvae entering the cave from the sinkholes seems unlikely due to the absence of any water source near the sinkholes.

### 4.2 Exit scenarios of Hyrcanian Goldenring from the cave

The main exit scenario for the larva is the exit from the water exit point (Figure 4). The main exit scenario for the newly emerged adults of the species is to exit from the cave entrance. According to the air flow in the cave from sinkholes toward the entrance, it may have happened, although it seems impossible. Also, when the newly emerged adults fly to another safe place in the streamside on the cave wall or other karstic structures, they can probably find the cave entrance and exit it. Furthermore, in newly emerged adult locations, the stream is deep (90 cm) with a high volume of water. Observation of an exuvia at 25 m from the entrance to 280 m due to the rivulet flow, attracting larvae to the light, and even the phenomenon of cave breathing, which occurs based on different temperature conditions and is known as the chimney effect (Gabrovšek, 2023) and also the direction of airflow of the cave in the warm season, it can be considered a chance to get out of the interior for the exit of adults.

### 4.3 Hunting strategies and natural history of *Cordulegaster* and related species in subterranean habitats

According to Suhling et al. (2015), migration of larvae along streams and rivulets and even out of water at night and hunting epigean on land have been mentioned in the *Cordulegaster* species. The *Cordulegaster* larvae in the cave can hunt for a variety of aquatic invertebrates. They can hunt other cave invertebrates even if they have fallen on water surface; in addition, cannibalism has been reported in this genus (Bo et al., 2011). Due to the darkness of the rivulet bed in the cave, the main hunting method of *C. vanbrinkae* larvae relies on the tactile method. In some research, in addition to hunting with the help of vision, hunting in the darkness with the help of mechanoreceptors has been mentioned, especially in *C. insignis*. The Mechanoreceptors are flattened, stiff, fan‐shaped setae located on the upper side of the head and forelegs (Corbet, 2004).

According to literature and evidence, these species tend toward darkness and shadow. Even the larvae of these species are observed in the Kariz (subterranean water canals; also known as kahriz and qanat), especially in the case of *C. charpentieri* in the deserts of central Iran, which are the only stable water resources for these areas. In such a habitat, larvae of other species, such as *Platycnemis dealbata*, have been observed, and adults frequently enter the underground canals of the Kariz (Kiany & Sadeghi, 2016).

There are several other species that have been recorded in caves around the world. Emperor Dragonfly, *Anax imperator* in Bosnia and Herzegovina (Ponikva Cave), Slovenia (Postojna Cave) and Hungary (Barado Cave) (Kiauta, 1995); Balkan Emerald, *Somatochlora meridionalis* in Italy and Slovenia (Zopenca Cave) (Kiauta, 1995); Sombre Goldenring or Two‐toothed Goldenring, *Cordulegaster bidentata* in France and Italy (Krieg‐Jacquier & Sansault, 2016; Manenti et al., 2013); Golden‐ringed Dragonfly *Cordulegaster boltonii* in France (Krieg‐Jacquier & Sansault, 2016); Australian Emerald, *Hemicordulia australiae* at the entrance of a cave during winter in New Zealand (Marinov, 2010); and Cave Duskhawker, *Gynacantha nourlangie* in Australia (Theischinger et al., 2021; Thompson & Kiauta, 1994) are good examples of the presence of dragonflies in at the entrance and in the twilight zones of caves and other subterranean habitats.

Our findings are important on a global and local scale. On a global scale, we represent a new chapter in cave biodiversity research and add a new order of invertebrates to the dark zone fauna in subterranean ecosystems, especially in natural riverine caves. Also, more studies are needed into the issue on a global scale, and we need to know whether more species are emerging in the dark zone or not. On a local scale, these records increase the importance of Danial Cave as a national natural monument (Ghelich Khani et al., 2023). Also, more studies on the penetration, entering ways and oviposition of the Hyrcanian Goldenring in the cave are needed. Likewise, the cave carrying capacity of the presence of visitors and even their absence at certain times needed. In addition, visitors to the cave must be trained, and their entry into the cave should be controlled.

## 5 CONCLUSION

There are some reports of Odonata species at the entrance and in the twilight zones of subterranean habitats worldwide. However, the emergence of Odonata species has not been recorded in the dark zone. The main hypothesis of the study is the entry and exit of adults of *Cordulegaster vanbrinkae* from the cave entrance. We believe the cave is the habitat of a small population of the species in the larval stage. The records are crucial on a global and local scale; on a global scale, a new order of invertebrates has been added to the dark zone fauna in the cave ecosystems. On a local scale, our findings increase the importance of Daniel Cave, which can be protected as a national natural monument and national heritage for the country. We still do not know if this habitat is permanent or temporary! We still do not know if the newly emerged adults leave the cave or not. More studies on the behavior of females around the cave and the oviposition of *C. vanbrinkae* inside the cave are needed.

## AUTHOR CONTRIBUTIONS

**Pouria Ghelich Khani:** Conceptualization (lead); data curation (lead); formal analysis (lead); funding acquisition (lead); investigation (lead); methodology (lead); project administration (lead); resources (lead); software (lead); supervision (lead); validation (lead); visualization (lead); writing – original draft (equal); writing – review and editing (equal). **Mohsen Kiany:** Conceptualization (equal); investigation (supporting); methodology (supporting); software (supporting); supervision (supporting); validation (equal); visualization (equal); writing – original draft (equal); writing – review and editing (supporting). **Ali Turk Qashqaei:** Investigation (supporting); methodology (supporting); project administration (supporting); software (supporting); supervision (supporting); validation (supporting); writing – original draft (equal); writing – review and editing (equal).

## FUNDING INFORMATION

None.

## CONFLICT OF INTEREST STATEMENT

We declare that there are no conflicts of interest.

## Data Availability

Data sharing is not applicable to this article as no new data were created or analyzed in this study.
